# The association between dietary acid load and muscle strength among Iranian adults

**DOI:** 10.1186/s13104-020-05309-6

**Published:** 2020-10-09

**Authors:** Saba Mohammadpour, Farhang Djafari, Samira Davarzani, Kurosh Djafarian, Cain C. T. Clark, Sakineh Shab-Bidar

**Affiliations:** 1 Department of Community Nutrition, School of Nutritional Sciences and Dietetics, Tehran University of Medical Sciences (TUMS), Iran. No 44, Hojjat-dost Alley, Naderi St., Keshavarz Blvd, Tehran, Iran; 2 Department of Clinical Nutrition, School of Nutritional Sciences and Dietetics, Tehran University of Medical Sciences (TUMS), Tehran, Iran; 3 Centre for Sport, Exercise, and Life Sciences, Coventry University, Coventry, CV15FB U.K.

**Keywords:** Dietary acid load, Muscle strength, Muscle mass, Acidogenic diet, Alkalogenic diet

## Abstract

**Objective:**

There is limited evidence regarding the association between dietary acid load and muscle strength. Thus, in this study, we investigated the association between dietary acid–base load indices and muscle strength among Iranian adults.

**Results:**

This cross-sectional study was conducted on 270 Iranian adults, aged 18–70 year. Dietary acid load indexes, were calculated by using a validated 168-item semi-quantitative food frequency questionnaire (FFQ). Muscle strength was measured by a digital handgrip dynamometer.

There was a significant increase in mean muscle strength of left-hand (MSL), muscle strength of right-hand (MSR) and the mean of the MSL and MSR (MMS) across tertiles of Potential Renal Acid Load (PRAL), Net Endogenous Acid Production (NEAP), and Dietary Acid Load (DAL). Significant linear relationships between PRAL and; MSL (*β* = 0.24, *p < *0.001), MSR (*β* = 0.23, *p < *0.001) and MMS (*β* = 0.24, *p < *0.001), between NEAP and MSL (*β* = 0.21, *p < *0.001), MSR (*β* = 0.19, *p = *0.002), and MMS (*β* = 0.20, *p = *0.001) and between DAL and MSL (*β* = 0.25, *p < *0.001), MSR (*β* = 0.23, *p < *0.001) and MMS (*β* = 0.24, *p* < 0.001), were attenuated after controlling for potential confounders. However, the nonlinear relationship between dietary acid load indicators and muscle strength were significant (p < 0.001 for all).

## Introduction

Muscle strength is defined as the ability to produce maximum force [1]. Muscle strength and muscle mass decline with aging; Comparatively, the decline is faster for muscle strength, and can lead to difficulties in daily activities, weakness, disability, decrease in quality of life, and early mortality [2]. Several factors, such as lifestyle, diet quality and dietary patterns [3], low protein intake [4], obesity [5] and physical activity [6] may elicit negative effects on muscle strength. Many nutritional studies that have investigated the effect of single macro- and micro-nutrients on skeletal muscle function have reported equivocal findings [7, 8]. For example, Daly et al. found that a diet enriched in protein from lean red meat (~ 1.3 gr/kg/day) can improve muscle strength in woman aged 60–90 year [7]; whilst a meta-analysis showed that supplemental protein intake improves muscle mass and strength just in resistance-trained individuals but not in trained ones [8].

Generally, foods rich in animal protein, such as meat, cheese, eggs, and grains increase the production of acid in the body because they are involved in the formation of hydrogen sulfate that arises from sulfur-containing amino acids, and phosphoric acid from phospholipids. In contrast, foods rich in plant protein and potassium, such as fruits and vegetables, increase alkaline products and are known as alkalogenic foods [9, 10]. Welch et al. reported a positive significant relationship between fat-free mass and an alkalogenic diet rich in fruits and vegetables [11]. Some formulas have been used to assess acid load from diet; for example, potential renal acid load (PRAL), net endogenous acid production (NEAP) [12], and dietary acid load (DAL) [13, 14]. PRAL and DAL scores are better predictors of dietary acid load, because NEAP is calculated from the ratio of protein and potassium, but PRAL and DAL formulas include calcium, phosphorus, and magnesium in addition to protein and potassium [9, 13].

To the best of our knowledge, no study has evaluated the link between dietary acid load and muscle strength. Therefore, our study aimed to investigate the relationship between dietary acid load and muscle strength among adults living in Iran.

## Main text

### Materials and methods

#### Study population

In this cross-sectional study, we enrolled 270 subjects who were living in Tehran, Iran. Recruitment was conducted via advertisement. Inclusion criteria included; age range of 18–70 year and were interested in participating in the study. Exclusion criteria included: (1) pregnant and lactating women, (2) any regular treatment with special supplements or drugs, (3) presence of clinical and subclinical diseases, and (4) inability to conduct tests. Trained research assistants collected information about health conditions and medical history. Information on physical activity, demographic data, also, blood pressure data, and anthropometric data were recorded.

#### Anthropometric measures and body composition

Height was measured to the nearest 0.1 cm using a wall-mounted stadiometer (Seca, Germany), and weight was measured to the nearest 0.1 kg using a digital scale (Seca 808, Germany), with both measurements taken with participants unshod and in light clothing. Body mass index (BMI) was calculated as weight in kilograms divided by height in meters squared. Waist circumference (WC) was measured, to the nearest 0.1 cm, midway between the inferior border of the rib cage and the superior aspect of the iliac crest, using a non-elastic tape. A body composition analyzer (In Body Sweden, 2017) was used for the measurement of body composition [15].

#### Blood pressure assessment

Blood pressure was measured using a digital sphygmomanometer (BC 08, Beurer, Germany). The participant sat for 10–15 min before two measurements were taken. The means for systolic blood pressure (SBP) and diastolic blood pressure (DBP) were used in subsequent analyses.

#### Physical activity

To assess physical activity, we used the short form of International Physical Activity Questionnaire (IPAQ) [16]. Data on moderate and vigorous activity, and walking for at least 10 min/day, during the previous 7 days were recorded according to the IPAQ criteria. Duration and frequency of the days of activity were multiplied by the activity's metabolic equivalent task value for calculating the activity. Total physical activity per week was used to define groups of low, moderate, and high, and weekly metabolic equivalent (METS)-Minutes were computed.

#### Dietary assessment

Dietary intake was calculated using a validated 168-item semi-quantitative food frequency questionnaire (FFQ) [17]. The participants reported their frequency of consumption of each food item during the previous year on a daily, weekly, or monthly basis. Nutrients were calculated using the Nutritionist IV software (First Databank, San Bruno, CA), modified for Iranian foods.

#### Assessment of dietary acid load

The measures of dietary acid–base load used were the PRAL, NEAP, and DAL indexes, which were calculated by using individual nutrients derived from the FFQ using the following formulas:

$$ {\text{PRAL}}\left( {{\text{mEq}}/{\text{day}}} \right){\text{ }}\, = \,\left( {0.{\text{49}}\, \times \,{\text{protein}}\left[ {{\text{g}}/{\text{day}}} \right]} \right)\, + \,\left( {0.0{\text{37}}\, \times \,{\text{phosphorus}}\left[ {{\text{mg}}/{\text{day}}} \right]} \right)\, - \,\left( {0.0{\text{21}}\, \times \,{\text{ potassium }}\left[ {{\text{mg}}/{\text{day}}} \right]} \right)\, - \,\left( {0.0{\text{26}}\, \times \,{\text{magnesium }}\left[ {{\text{mg}}/{\text{ day}}} \right]} \right)\, - \,\left( {0.0{\text{13}}\, \times \,{\text{calcium }}\left[ {{\text{mg}}/{\text{day}}} \right]} \right) $$

^18^]

$$~{\text{NEAP}}\left( {{\text{mEq}}/{\text{day}}} \right)\, = \,{\text{54}}.{\text{5}}\, \times \,{\text{protein}}\left( {{\text{g}}/{\text{day}}} \right)\, \div \,{\text{potassium}}\left( {{\text{mg}}/{\text{day}}} \right)\, - \,{\text{1}}0.{\text{2}} $$

[9]

$$ {\text{DAL}}\left( {{\text{mEq}}/{\text{ day}}} \right)\, = \,{\text{PRAL}}\, + \,\left( {{\text{body surface area }}\left[ {{\text{m}}^{{\text{2}}} } \right]\, \times \,{\text{41 }}\left[ {{\text{mEq }}/{\text{ day}}} \right]/{\text{1}}.{\text{73 m}}^{{\text{2}}} } \right) $$

[19]

Body surface area was calculated using the Du Bois formula: $0.00{\text{7184}}\, \times \,{\text{height }}0.{\text{725}}\, \times \,{\text{weight }}0.{\text{425}}$ [13, 14]

To calculate PRAL and NEAP, intake data were required for protein, phosphorus, potassium, calcium, and magnesium. PRAL, DAL, and NEAP scores were derived from the equations of nutrient intakes, and tertiles of the scores were used for statistical analysis.

#### Muscle strength

Muscle strength was measured by a digital handgrip dynamometer (Saehan, model SH5003; Saehan Corporation, Masan, South Korea). The forearm and wrist of participants were required to be in a normal position, the dynamo-meter grip size was set to the size of their hands; participants were then asked to squeeze the dynamometer handle as hard as possible to exert maximum force. The procedure was repeated three times with each hand, and in total, handgrip strength was measured 6 times. For data analysis, the mean grip strength was calculated using the best attempt from each hand [20].

#### Statistical analyses

All statistical analyses were performed using The Statistical Package for the Social Sciences (SPSS version 14.) version 25. We analyzed the study participants ‘characteristics according to PRAL, NEAP, and DAL tertiles, using one-way analysis of variance (ANOVA) to compare continuous variables and χ^2^ tests for categorical variables. ANOVA was used to compare muscle strength across tertiles of the PRAL, NEAP, and DAL. We then used analysis of covariance (ANCOVA) to adjust for potentially confounding variables, such as age, sex, education, occupation, marriage, living situation, smoking status, fat mass, physical activity, meat consumption, and energy intake. Linear and non-linear regression models were fitted to assess the effect of dietary acid load indicators on muscle strength. Statistical significance was accepted at p < 0.05.

#### Results

The general characteristics of participants, across the tertiles of PRAL, NEAP, and DAL, are shown in Table 1. The mean of age and fat-free mass was significantly decreased across tertiles of PRAL, NEAP, and DAL. However, systolic blood pressure was higher in the third tertile of NEAP compared to the first tertile. Those who had greater adherence to all three indices were; married, male, educated, employed, smokers, and had better living situation and metabolic markers.

**Table 1:** Characteristics of participants by tertiles (T) of PRAL, NEAP and DAL

	PRAL							NEAP							DAL						
	T1 (1–89)		T2 (90–179)		T3 (180–268)		p	T1 (1–89)		T2 (90–179)		T3 (180–268)		p	T1 (1–89)		T2 (90–179)		T3 (180–268)		p
Participants	89		90		89			89		90		89			89		90		89		
Characteristics	Mean	SD	Mean	SD	Mean	SD		Mean	SD	Mean	SD	Mean	SD		Mean	SD	Mean	SD	Mean	SD	
Age (y)	41.8	13.9	34.9	11.7	33.0	12.0	< 0.001	41.7	14.0	34.2	11.3	33.8	12.4	< 0.001	41.8	13.9	34.6	11.7	33.2	12.0	< 0.001
Weight (kg)	71.7	14.7	71.4	17.8	75.1	15.4	0.22	71.6	14.6	70.0	17.2	76.6	15.3	0.01	70.7	14.5	72.1	18.0	75.3	15.3	0.14
BMI (kg/m^2^)	26.1	4.78	24.9	4.43	25.7	4.81	0.21	26.0	4.42	24.7	4.79	26.1	4.76	0.08	25.9	4.87	25.1	4.38	25.7	4.81	0.52
WC (cm)	81.8	11.8	88.6	13.1	90.3	12.9	0.62	89.8	11.3	87.4	13.5	91.6	12.6	0.07	89.2	11.8	89.1	13.1	90.4	12.8	0.74
WHR (cm)	0.90	0.05	0.89	0.06	0.90	0.07	0.83	0.90	0.05	0.89	0.06	0.91	0.06	0.13	0.90	0.05	0.90	0.06	0.90	0.07	0.94
Fat-mass (kg)	23.9	9.44	21.8	8.71	21.6	10.0	0.20	23.7	8.60	21.4	9.64	22.2	9.93	0.24	23.7	9.51	22.0	8.64	21.5	10.0	0.28
Fat-free mass (kg)	47.8	11.1	49.5	12.7	53.1	13.5	0.01	47.8	11.4	48.5	11.7	54.0	13.8	0.001	47.0	10.5	50.0	13.0	53.4	13.5	0.03
SBP (mmHg)	114	14.5	106	27.3	113	10.1	0.05	112	18.2	105	24.1	116	11.5	0.001	114	14.3	106	27.5	113	10.1	0.008
DBP (mmHg)	70.8	11.4	70.7	12.5	70.3	7.60	0.96	70.3	11.2	69.0	11.3	72.6	9.11	0.07	70.7	11.2	70.7	12.6	70.4	7.57	0.98
	(%)																				
Sex							< 0.001							< 0.001							< 0.001
Male	30.3		41.1		59.6			30.3		37.8		62.9			28.1		42.2		60.7		
Female	69.7		58.9		40.4			69.7		62.2		37.1			71.9		57.8		39.3		
Education							0.03							0.12							0.03
Illiterate	0.0		0.0		1.1			0.0		0.0		1.1			0.0		0.0		1.1		
Under diploma	14.6		5.6		2.2			12.4		6.7		3.4			14.6		5.6		2.2		
Diploma	21.3		17.8		16.9			22.5		18.9		14.6			21.3		17.8		16.9		
Educated	64.0		76.7		79.8			65.2		74.4		80.9			64.0		76.7		79.8		
Occupation							< 0.001							< 0.001							< 0.001
Employee	41.6		61.1		57.3			43.8		54.4		61.8			40.4		61.1		58.4		
Housekeeper	31.5		7.8		10.1			28.1		13.3		7.9			31.5		7.8		10.1		
Retired	13.5		4.4		5.6			15.7		2.2		5.6			13.5		4.4		5.6		
Unemployed	13.5		4.4		5.6			12.4		30.0		24.7			14.6		26.7		25.8		
Marriage							0.03							< 0.001							0.003
Single	25.8		47.8		55.1			24.7		45.6		58.4			25.8		48.9		53.9		
Married	67.4		50.0		41.6			68.5		50.0		40.4			67.4		48.9		42.7		
Divorced	3.4		2.2		2.2			3.4		4.4		0.0			3.4		2.2		2.2		
Dead spouse	3.4		0.0		0.0			3.4		0.0		0.0			3.4		0.0		0.0		
Living situation							0.02							0.001							0.02
Alone	4.5		6.7		15.7			3.4		5.6		18.0			4.5		6.7		15.7		
With someone	95.5		93.3		84.3			96.6		94.4		82.0			95.5		93.3		84.3		
Smoking																					
Not smoking	93.3		4.5		2.2		0.04	92.1		5.6		2.2		0.03	93.3		4.5		2.2		0.04
Quit smoking	87.8		4.4		7.8			88.9		2.2		8.9			87.8		4.4		7.8		
Smoking	78.7		6.7		14.6			78.7		7.9		13.5			78.7		6.7		14.6		
Metabolic disorders^b^																					
No	74.2		86.7		88.6		0.02	77.5		87.8		84.1		0.17	74.2		86.7		88.6		0.02
Yes	25.8		13.3		11.4			22.5		12.2		15.9			25.8		13.3		11.4		
Activity score^c^																					
Low	39.3		40.0		34.8		0.64	46.1		36.7		31.5		0.06	40.4		40.0		33.7		0.35
Moderate	43.8		41.1		39.3			41.6		43.3		39.3			44.9		40.0		39.3		
High	16.9		18.9		25.8			12.4		20.0		29.2			14.6		20.0		27.0		

*PRAL* Potential renal acid load, *NEAP* Net endogenous acid production, *DAL* Dietary acid load, *BMI* body mass index, *WHR* waist-hip ratio, *WC* waist circumference, *SBP* systolic blood pressure, *DBP* diastolic blood pressure, *TC* total cholesterol

P values result from ANOVA for quantitative variables and χ^2^ test for qualitative variables

^a^Values are means ± SD

^b^Metabolic disorders: Including the history of diabetes, hypertension, dyslipidemia, cardiovascular disease, stroke, cancer, respiratory disease and osteoporosis

^c^Activity score: Data on vigorous and moderate activity and walking for at least 10 min/day during the previous 7 days were recorded according to the IPAQ criteria. Duration and frequency of the days of activity were multiplied by the activity's metabolic equivalent task value for calculating the activity. The total physical activity per week was used to measure the sum of the scores divided into three groups: low, moderate, and high. IPAQ was also measured for a continuous score and stated as an equivalent metabolic (MET)-minutes per week.

Dietary intake of participants for each category of PRAL, NEAP, and DAL are detailed in Additional file 1: Table S1. With increasing tertiles of PRAL, NEAP, and DAL, the mean total energy, grains, red meat, white meat and fish, protein, carbohydrate, and total fat, increased, whilst intake of fruits, vegetables, and potassium decreased.

As detailed in Table 2, in the crude model, there was a significant increase in mean muscle strength of left-hand (MSL), muscle strength of right-hand (MSR), and the mean of the MSL and MSR (MMS) across tertiles of PRAL, NEAP, and DAL. However, after adjustment for confounders, the significant differences were attenuated.

**Table 2:** Muscle strength by tertiles (T) of PRAL, NEAP and DAL

	PRAL							NEAP							DAL						
	T1	T2	T3	P	P1	P2	P3	T1	T2	T3	P	P1	P2	P3	T1	T2	T3	P	P1	P2	P3
	Mean ± SD	Mean ± SD	Mean ± SD					Mean ± SD	Mean ± SD	Mean ± SD					Mean ± SD	Mean ± SD	Mean ± SD				
MSL (kg)	27.5 ± 10.4	28.5 ± 11.1	33.3 ± 12.2	0.001	0.22	0.22	0.28	27.2 ± 10.0	28.5 ± 11.1	33.6 ± 12.4	< 0.001	0.79	0.80	0.83	26.9 ± 9.88	28.9 ± 11.4	33.6 ± 12.2	< 0.001	0.31	0.32	0.36
MSR (kg)	30.1 ± 11.6	31.0 ± 11.7	35.9 ± 12.7	0.003	0.24	0.25	0.30	30.1 ± 12.0	30.8 ± 11.2	36.1 ± 12.7	0.002	0.54	0.54	0.54	29.4 ± 11.1	31.4 ± 11.9	36.2 ± 12.7	0.001	0.35	0.36	0.43
MMS (kg)	28.8 ± 10.8	29.8 ± 11.2	34.6 ± 12.2	0.001	0.20	0.21	0.26	28.7 ± 10.8	29.6 ± 11.0	34.8 ± 12.4	0.001	0.66	0.66	0.68	28.1 ± 10.3	30.1 ± 11.5	34.9 ± 12.3	< 0.001	0.30	0.31	0.36

P1: Adjusted for age, sex, education, occupation, marriage, Living situation, smoking, fat mass and physical activity

P2: Adjusted for age, sex, education, occupation, marriage, Living situation, smoking, fat mass, physical activity and red meat

P3: Adjusted for age, sex, education, occupation, marriage, Living situation, smoking, fat mass, physical activity, red meat and energy intake

*PRAL* Potential renal acid load, *NEAP* Net endogenous acid production, *DAL* Dietary acid load, *MSL* muscle strength of left hand, *MSR* muscle strength of right hand; MMS, mean Muscle strength

There were significant relationships between PRAL and MSL (β = 0.24, p < 0.001), MSR (β = 0.23, p < 0.001), and MMS (β = 0.24, p < 0.001). Also, significant associations between NEAP and MSL (β = 0.21, p < 0.001), MSR (β = 0.19, p = 0.002), and MMS (β = 0.20, p = 0.001) were observed. Moreover, we found significant relationships between DAL and MSL (β = 0.25, p < 0.001), MSR (β = 0.23, p < 0.001), and MMS (β = 0.24, p < 0.001). After controlling for potential confounders, there was no significant association (Additional file 1: Table S2).

**Fig. 1:**
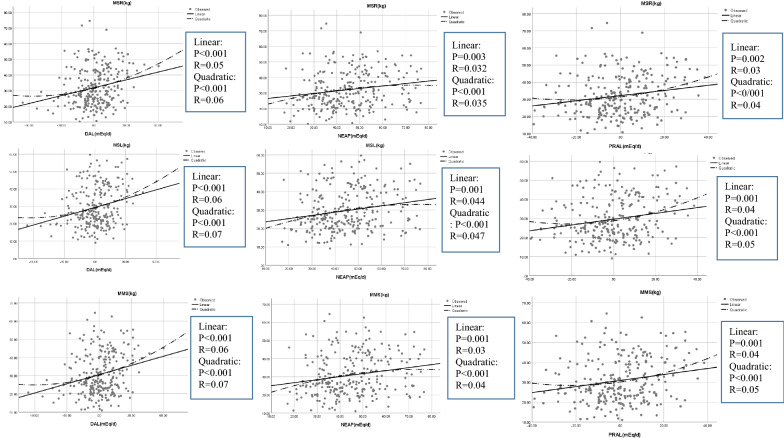
Association of dietary acid load indices and muscle strength

There was a significant nonlinear relationship between dietary acid load indicators and muscle strength (p < 0.001), where muscle strength increased with increasing dietary acid load indicators (Fig. 1).

### Discussion

In the present study, we found a significant positive association between indicators of dietary acid load and muscle strength. However, these associations were not significant after adjustment for confounding variables. In non-linear regression model, a higher dietary acid load was associated with greater muscle strength.

Higher consumption of foods rich in animal protein, such as meat, cheese, eggs, and, grains increases the production of acid in the body. This study indicates that with increasing dietary acid load, intakes of grains, dairy, red meat, and white meat and fish were increased. They are associated with the production of hydrogen sulfate that arises from sulfur-containing amino acids, and phosphoric acid from phospholipids, which consequently increases dietary acid load. Consistent with our results, Masuki et al. inferred that a high intake of milk products after exercise yielded a significant increase in thigh muscle strength [21]; Contrastingly, Alemán-Mateo et al. in a randomized clinical trial of sarcopenic elderly men and women over 60 years, showed that adding 210 g/day of ricotta cheese to their habitual diet yielded no significant change in total appendicular skeletal muscle. It appears that the amount of protein intake may have an effect on sarcopenic status in the elderly. However, in Alemán-Mateo et al. handgrip strength improved following consumption of protein-rich foods, which suggests that the loss of skeletal muscle in elderly men may be reversed, or ameliorated, by adding protein-rich foods, such as ricotta cheese, to the diet [22].

In our study, the intake of meat increased across tertiles of PRAL, NEAP, and DAL. Red meat is a high-quality source of protein containing some compounds that can influence protein metabolism, such as some minerals (iron and zinc), vitamins (mainly B group vitamins) [23], creatine [24], carnitine, carnosine, and conjugated linoleic acid [25], in addition, it also contains complete sufficient essential amino acids to promote the synthesis of skeletal muscle proteins, and to preserve skeletal muscle mass [26, 27]. In line with our findings, Symons et al. asserted that a moderate intake of lean meat can improve synthesis of protein in both genders [28]. In contrast, in a sample of older adults, Granic et al. reported that dietary patterns high in red meat, would adversely influence muscle strength [29]. It has been putatively hypothesized that this may be related to the impact of adverse acid–base equilibrium on muscle strength [30, 31], and the adverse effects of arachidonic acid (AA) on muscle size, due to increased expression and activation of the ubiquitin–proteasome pathway, which may trigger muscle protein degradation [32]. It is conceivable that our results may be influenced by the mediating effect of protein. Morton RW, et al. in a meta-analysis, concluded that post-exercise protein intake, combined with resistance exercise training (RET), can lead to increases in muscle mass, fat-free mass, muscle size, and strength [8]. Indeed, empirical studies have suggested that such improvements may be due to the effects of protein on muscle hypertrophy and function, whilst it has also been reported that ingestion of 20–30 g or 0.25–0.30 g/kg protein after resistance exercise training, with habitual protein intakes at ~ 1.6 g/kg/day stimulates muscle adaptations to exercise training [8].

In contrast with our findings, Neville et al. found that greater fruit and vegetable consumption can yield increases grip strength, however, the period of their study was short [33]. Dawson-Hughes et al. also found that higher alkaline dietary load, is positively associated with muscle mass indices [30]; however, this association was weak, and after controlling for the percentage of protein intake, became weaker.

The exact mechanism through which an alkalotic diet can affect muscle mass and strength is not completely understood. Studies suggest that, although protein is essential for muscle mass conservation [34, 35], other nutrients in the alkalinogenic foods, included potassium, magnesium [33, 36], antioxidants, and carotenoid content, may protect against oxidative stress and inflammation [37, 38].

### Conclusion

we found a significant positive association between a higher dietary acid loads and greater muscle strength. Further studies are needed to confirm the veracity of our findings.

## Limitations

As with any cross-sectional study design, no causal associations can be determined, and although we adjusted for possible confounders, we cannot exclude the possibility of residual confounding variables.

## Supplementary information

**Additional file 1: Table S1.** Dietary intakes of participants by tertiles(T) of PRAL, NEAP and DAL.** Table S2.** Multiple linear regression between muscle strength and indexes of dietary acid load.

## Data Availability

Since the privacy of research participants may be compromised, we cannot make the information publicly available.

## Abbreviations

PRAL: Potential renal acid load
NEAP: Net endogenous acid production
DAL: Dietary acid load
MSL: Mean muscle strength of left-hand
MSR: Muscle strength of right-hand
MMS: Mean muscle strength
CVD: Cardiovascular disease
CKD: Chronic kidney disease
WC: Waist circumference
SBP: Systolic blood pressure
DBP: Diastolic blood pressure
FBS: Fasting blood glucose
HDL-C: High-density lipoprotein
TG: Triglyceride
IPAQ: International physical activity questionnaire
FFQ: Food frequency questionnaire
ANOVA: Analysis of variance
SFA: Saturated fatty acid
MUFA: Monounsaturated fatty acid
AA: Arachidonic acid
SMM: Skeletal muscle mass
TNF-α: Tumor necrosis factor*-*α
