# Supplementary material for: The association between dietary acid load and muscle strength among Iranian adults
Source: BMC Res Notes. 2020 Oct 9;13:476. doi: 10.1186/s13104-020-05309-6 (PMC7547515; doi:10.1186/s13104-020-05309-6)
Supplement: Supplementary file 1 — Additional file 1: Table S1. Dietary intakes of participants by tertiles(T) of PRAL, NEAP and DAL. Table S2. Multiple linear regression between muscle strength and indexes of dietary acid load. [file 13104_2020_5309_MOESM1_ESM.docx]

**Table S1:** Dietary intakes of participants by tertiles(T) of PRAL, NEAP and DAL

|  | PRAL | | | | | |  | NEAP | | | | | |  | DAL | | | | | |  |
| --- | --- | --- | --- | --- | --- | --- | --- | --- | --- | --- | --- | --- | --- | --- | --- | --- | --- | --- | --- | --- | --- |
|  | T1  (1-89) | | T2  (90-179) | | T3  (180-268) | | P | T1  (1-89) | | T2  (90-179) | | T3  (180-268) | | P | T1  (1-89) | | T2  (90-179) | | T3  (180-268) | | P |
| Participants | 89 | | 90 | | 89 | |  | 89 | | 90 | | 89 | |  | 89 | | 90 | | 89 | |  |
|  | Mean | SD | Mean | SD | Mean | SD |  | Mean | SD | Mean | SD | Mean | SD |  | Mean | SD | Mean | SD | Mean | SD |  |
| Total calorie intake (kcal) | 2192 | 775 | 2185 | 749 | 2797 | 1186 | <0.001 | 2177 | 718 | 2267 | 793 | 2729 | 1223 | <0.001 | 2180 | 778 | 2184 | 761 | 2809 | 1170 | <0.001 |
| Grains (gr/d) | 414 | 169 | 488 | 193 | 582 | 261 | <0.001 | 413 | 156 | 489 | 205 | 581 | 261 | <0.001 | 412 | 169 | 487 | 194 | 584 | 259 | <0.001 |
| Fruits (gr/d) | 407 | 201 | 232 | 131 | 261 | 172 | <0.001 | 397 | 205 | 252 | 141 | 250 | 169 | <0.001 | 404 | 202 | 234 | 133 | 262 | 171 | <0.001 |
| Vegetable (gr/d) | 460 | 243 | 300 | 170 | 264 | 183 | <0.001 | 449 | 242 | 305 | 165 | 271 | 199 | <0.001 | 456 | 243 | 303 | 175 | 266 | 182 | <0.001 |
| Red meat (gr/d) | 35.3 | 28.5 | 38.3 | 22.3 | 60.8 | 51.2 | <0.001 | 31.8 | 22.5 | 42.9 | 27.8 | 59.6 | 51.4 | <0.001 | 34.4 | 28.0 | 38.6 | 22.7 | 61.4 | 50.9 | <0.001 |
| White meat and Fish (gr/d) | 48.1 | 35.6 | 51.3 | 32.8 | 117 | 93.8 | <0.001 | 45.4 | 35.5 | 56.3 | 33.1 | 115 | 95.1 | <0.001 | 47.2 | 35.0 | 52.3 | 33.3 | 117 | 93.9 | <0.001 |
| Red and white meat (gr/d) | 108 | 57.6 | 117 | 60.5 | 218 | 118 | <0.001 | 101 | 49.8 | 132 | 73.3 | 210 | 119 | <0.001 | 105 | 53.9 | 119 | 63.4 | 218 | 118 | <0.001 |
| Dairy (gr/d) | 465 | 341 | 419 | 313 | 506 | 275 | 0.17 | 470 | 315 | 442 | 324 | 478 | 298 | 0.72 | 459 | 342 | 419 | 313 | 512 | 272 | 0.13 |
| Protein (gr/d) | 77.8 | 31.3 | 76.5 | 26.5 | 123 | 75.2 | <0.001 | 75.6 | 27.7 | 82.6 | 31.7 | 119 | 76.7 | <0.001 | 77.1 | 31.2 | 76.8 | 27.1 | 124 | 74.7 | <0.001 |
| Carbohydrate (gr/d) | 325 | 117 | 310 | 111 | 383 | 190 | 0.002 | 324 | 108 | 315 | 119 | 379 | 192 | 0.006 | 324 | 117 | 309 | 113 | 385 | 188 | 0.001 |
| Total fat (gr/d) | 71.3 | 34.3 | 76.2 | 34.5 | 89.7 | 39.2 | 0.002 | 71.2 | 33.8 | 80.4 | 34.1 | 85.6 | 40.9 | 0.02 | 70.9 | 34.0 | 76.1 | 35.1 | 90.2 | 38.7 | 0.001 |
| Potassium (mg/d) | 4021 | 1548 | 3019 | 1118 | 3671 | 1859 | <0.001 | 3980 | 1501 | 3247 | 1261 | 3482 | 1871 | 0.007 | 3990 | 1559 | 3032 | 1148 | 3690 | 1842 | <0.001 |
| Calcium (mg/d) | 1026 | 513 | 892 | 438 | 1186 | 637 | 0.001 | 1022 | 490 | 970 | 467 | 1110 | 660 | 0.22 | 1017 | 514 | 892 | 443 | 1195 | 629 | 0.001 |
| Magnesium (mg/d) | 299 | 113 | 250 | 87.2 | 317 | 153 | 0.001 | 298 | 107 | 267 | 99.4 | 300 | 155 | 0.14 | 297 | 113 | 250 | 88.7 | 318 | 151 | 0.001 |
| Phosphorus  (mg/d) | 1244 | 547 | 1140 | 471 | 1619 | 894 | <0.001 | 1265 | 556 | 1236 | 491 | 1500 | 925 | 0.02 | 1232 | 548 | 1143 | 475 | 1627 | 888 | <0.001 |
| PRAL, Potential renal acid load; NEAP, Net endogenous acid production; DAL, Dietary acid load  * Values are means ± SD, P values result from ANOVA | | | | | | | | | | | | | | | | | | | | | |

**Table S2:** Multiple linear regression between muscle strength and indexes of dietary acid load

|  | PRAL | | | | NEAP | | | | DAL | | | | |
| --- | --- | --- | --- | --- | --- | --- | --- | --- | --- | --- | --- | --- | --- |
|  | β | R2 | CI | P | β | R2 | CI | P | β | R2 | CI | P |  |
| Crude |  |  |  |  |  |  |  |  |  |  |  |  |  |
| MSL (kg) | 0.24 | 0.06 | 0.06-0.17 | <0.001 | 0.21 | 0.04 | 0.07-0.24 | <0.001 | 0.25 | 0.06 | 0.06-0.18 | <0.001 |  |
| MSR (kg) | 0.23 | 0.05 | 0.05-0.18 | <0.001 | 0.19 | 0.03 | 0.05-0.24 | 0.002 | 0.23 | 0.05 | 0.06-0.18 | <0.001 |  |
| MMS (kg) | 0.24 | 0.05 | 0.06-0.17 | <0.001 | 0.20 | 0.04 | 0.06-0.24 | 0.001 | 0.24 | 0.06 | 0.06-0.18 | <0.001 |  |
| Model 1 |  |  |  |  |  |  |  |  |  |  |  |  |  |
| MSL (kg) | -0.003 | 0.63 | -0.04 -0.04 | 0.95 | 0.007 | 0.63 | -0.05-0.06 | 0.87 | - 0.001 | 0.63 | -0.04 - 0.04 | 0.97 |  |
| MSR (kg) | -0.02 | 0.63 | -0.06 - 0.03 | 0.57 | -0.01 | 0.63 | -0.07-0.04 | 0.65 | -0.02 | 0.63 | -0.06 - 0.03 | 0.59 |  |
| MMS (kg) | -0.01 | 0.81 | -0.05 - 0.03 | 0.74 | -0.006 | 0.65 | -0.06-0.05 | 0.87 | -0.01 | 0.65 | -0.05 - 0.03 | 0.76 |  |
| PRAL, Potential renal acid load; NEAP, Net endogenous acid production; DAL, Dietary acid load  MSL, muscle strength of left hand; MSR, muscle strength of right hand; MMS, mean muscle strength.  Model 1: adjusted for age, sex, occupation, Living situation, smoking, BMI, physical activity and energy intake | | | | | | | | | | | | |  |
